# An Efficient Algorithm to Perform Local Concerted Movements of a Chain Molecule

**DOI:** 10.1371/journal.pone.0118342

**Published:** 2015-03-31

**Authors:** Stefano Zamuner, Alex Rodriguez, Flavio Seno, Antonio Trovato

**Affiliations:** 1 Dipartimento di Fisica e Astronomia G. Galilei, Universitá degli Studi di Padova, Via Marzolo 8, I-35131 Padova, Italy; 2 SISSA (Scuola Internazionale Superiore di Studi Avanzati), via Bonomea 265, I-34136 Trieste, Italy; 3 INFN (Istituto Nazionale di Fisica Nucleare), Sezione di Padova, Via Marzolo 8, I-35131 Padova, Italy; 4 CNISM (Consorzio Nazionale Interuniversitario per le Scienze fisiche della Matera), Unità di Padova, Via Marzolo 8, I-35131 Padova, Italy; Jacobs University Bremen, GERMANY

## Abstract

The devising of efficient concerted rotation moves that modify only selected local portions of chain molecules is a long studied problem. Possible applications range from speeding the uncorrelated sampling of polymeric dense systems to loop reconstruction and structure refinement in protein modeling. Here, we propose and validate, on a few pedagogical examples, a novel numerical strategy that generalizes the notion of concerted rotation. The usage of the Denavit-Hartenberg parameters for chain description allows all possible choices for the subset of degrees of freedom to be modified in the move. They can be arbitrarily distributed along the chain and can be distanced between consecutive monomers as well. The efficiency of the methodology capitalizes on the inherent geometrical structure of the manifold defined by all chain configurations compatible with the fixed degrees of freedom. The chain portion to be moved is first opened along a direction chosen in the tangent space to the manifold, and then closed in the orthogonal space. As a consequence, in Monte Carlo simulations detailed balance is easily enforced without the need of using Jacobian reweighting. Moreover, the relative fluctuations of the degrees of freedom involved in the move can be easily tuned. We show different applications: the manifold of possible configurations is explored in a very efficient way for a protein fragment and for a cyclic molecule; the “local backbone volume”, related to the volume spanned by the manifold, reproduces the mobility profile of all-α helical proteins; the refinement of small protein fragments with different secondary structures is addressed. The presented results suggest our methodology as a valuable exploration and sampling tool in the context of bio-molecular simulations.

## Introduction

We consider the problem of the local movements of a chain molecule where a small subset of degrees of freedom, e.g. dihedral angles, bonds angles or bond lengths, are concertedly modified inside a specific portion of the chain, in such a way that *only* the atoms in that region are moved while all the others are kept fixed. We do not place any constraints on the degrees of freedom that are modified: they can be chosen everywhere along the part of the chain we want to move without the necessity of belonging to atoms/bonds which are consecutive along the polymer. The issue of local movements is related to the loop closure problem, i.e. finding conformations of a segment of consecutive atoms in a chain molecule that are geometrically consistent with the rest of the chain structure. These questions arise in the context of the control of robotic manipulators made up of serially connected joints, where in many common applications one end is fixed and the other must be positioned at a specific location and with a given orientation [1], but they are also topics of paramount importance in structural chemistry and in computational biology. For instance, effective loop closure tools can enhance the performances of homology modeling where segments of insertion or deletion have to be predicted while the rest of the protein structure is reasonably well known from structures of homologous proteins. The ability of moving efficiently a chain may as well have useful applications in the Monte Carlo dynamics for large scale simulations of dense polymeric systems [2]. In such situation, the efficiency of Monte Carlo simulations relies heavily on the kinetic algorithm used to sample the various possible conformational states of the molecule and the introduction of a concerted move which restricts itself to modifying atom locations only in limited positions of the molecule might play a key role to boost performances, by reducing the hindering effect due to excluded volume constraint. Even the theoretical study of conformational flexibility can benefit from the use of local collective movements that, at variance with Cartesian moves, avoid geometric distorsions of the chain giving the possibility to explore all the possible local arrangements of the flexible molecule.

In the biological context, the problem was often reduced to modify dihedral angles that are the only soft degrees of freedom of the system. A first analysis was due to Go and Scheraga [3] with an analytical approach and with the developing of equations for determining the allowed dihedral angles when all the rotating bonds are connected and when a subset is separated by rigid bonds in the trans conformation. This approach was further extended by Theodoru and coworkers [4] to take into account the necessary requirements to ensure Boltzmann distributed sampling for Monte Carlo simulations and by Dinner [5] to generalize the formalism to allow fixed dihedral angles that sequentially interrupt the rotating bonds to be non-planar. Another refinement was later proposed with methods [6–9] in which the inclusion of bond angle variations and of local constraints improved the efficiency of the algorithm. Different formalisms were proposed by Hoffmann and Knapp [10] to derive equations for dihedral angles and to apply these moves to study a protein-like model that had the topology of polyalanine with rigid peptide planes and by Coutsias et al. [11, 12] with a robotics inspired approach. The latter approach was succesfully incorporated in state-of-art protein modeling tools, allowing sub-angstrom accuracy in loop reconstruction [13, 14]. More recently, an efficient numerical method to solve the analytical solution of the classic chain closure problem was introduced with the specific purpose of optimizing Monte Carlo performances for dense molecular systems [15].

Broadly speaking, all these methods are proposing efficient solutions, while violating the general rules of the problem: either by imposing restrictive conditions on the degrees of freedom that can be used (e.g. moving only specific angles) or by relaxing the boundary constraints (e.g. not keeping completely fixed all the other degrees of freedom).

On the contrary, in this paper we develope a numerical strategy that, exploiting the knowledge of an initial configuration of the chain, allows for an exhaustive exploration of all the possible configurations that can be obtained by modifying *only* *n* > 6 degrees of freedom, and that perfectly adapts to the frozen part of the chain. The choice of the degrees of freedom themselves is completely free and any combination of bond and dihedral angles and of bond lengths can be selected, resulting in a very rapid and efficient search algorithm.

Starting from a geometrical description of the chain inspired by robotic language [16], similar in spirit to the one introduced by Go and Scheraga [3], we derive six numerical equations, as a function of the *n* free variables, which have to be fulfilled to satisfy the boundary conditions. Therefore, if there is no degeneracy, the solutions that have to be found lie on a manifold with dimension *n* − 6. The novel idea we here present consists in an extremely powerful strategy to explore these manifolds, based on moving slightly out along their tangent space and on coming back along the orthogonal space towards a new configuration which satisfies all the equations and constraints. This is viable by means of an appropriate double change of coordinates and by employing mathematical algorithms to optimize the computational time. Moreover, the algorithm is designed in such a way that the detailed balance is quite easily satisfied for a very general choice of the modified degrees of freedom.

The efficiency of the approach is remarkable and it makes possible, for instance, to estimate the volume of the manifold which corresponds to the number of possible conformations that are compatible with the constraints, in the simplest *n* = 7 case. While at this stage the method is presented for an ideal chain, without taking into account excluded volume or other energy functions, such features can be introduced in a straightforward manner.

Although the method is completely general and can be applied to any sort of linear object, it is intriguing to think about its applications to protein chains. In such context bond angles and bond lengths can be considered constant and the *ψ* and *ϕ* dihedral angles (Ramachandran’s angles) are the natural degrees of freedom to be modified: the algorithm we propose thus becomes a generalized crankshaft move involving a portion of the chain of desired length. Some possible applications on proteins are shown, such as the estimation of their backbone mobilities and local structure refinement.

## Materials and Methods

### Denavit-Hartenberg paramaters for chain description

In this section we introduce the parametric representation of a linear chain used to derive the equations at the core of our algorithm. We consider a linear chain composed of *N* + 1 atoms linked serially in which each of the *N* bonds can be labeled with numbers from 1 to *N*. We describe the chain by using the Denavit-Hartenberg (DH) notation [17]. According to DH a local reference system 𝒪_*i*_ can be built on each bond composing the chain: the *ẑ*_*i*_ axis lies on the bond while *x̂*_*i*_ is oriented as *ẑ*_*i*−1_ × *ẑ*_*i*_. The *ŷ*_*i*_ axis is given, as usual, by the right-hand rule. The origin *o*_*i*_ of each reference frame is always located along *ẑ*_*i*_: in case *ẑ*_*i*_ is co-planar to *ẑ*_*i*−1_, it lies on the first atom defining the bond; otherwise it lies on the common normal to *ẑ*_*i*_ and *ẑ*_*i*−1_.

A vector ${\vec{a}}^{j}$ in the reference frame 𝒪_*j*_ can be expressed relative to 𝒪_*j*−1_ as

$$\begin{array}{c} {\vec{a}}^{j-1}=R_{j}^{j-1}\cdot {\vec{a}}^{j}+{\vec{S}}_{j}^{j-1}, \end{array}$$ (1)

where ${\text{R}}_{j}^{j-1}$ is a 3 × 3 orthogonal matrix that expresses the orientation of 𝒪_*j*_ relative to 𝒪_*j*−1_ and ${\vec{S}}_{j}^{j-1}$ is a vector describing the position of the origin *o*_*j*_ with respect to 𝒪_*j*−1_. The matrix ${\text{R}}_{j}^{j-1}$ and the vector ${\vec{S}}_{j}^{j-1}$ can be completely described by using four parameters named *link offset*, *link twist*, *link length* and *joint angle*. These are defined by the following rules:

- the *link offset* *d*_*i*_ is the distance along *x̂*_*i*_ from *o*_*i*_ to the intersection of the *x̂*_*i*_ and the *ẑ*_*i*−1_ axes (*i.e*. the minimum distance between *ẑ*_*i*−1_ and *ẑ*_*i*_ axis);
- the *link twist* *α*_*i*_ is the angle between *ẑ*_*i*−1_ and *ẑ*_*i*_ measured about *x̂*_*i*_;
- the *link length* *r*_*i*_ is the distance along *ẑ*_*i*−1_ from *o*_*i*−1_ to the intersection of the *x̂*_*i*_ and the *ẑ*_*i*−1_ axes and
- the *joint angle* *θ*_*i*_ is the angle between *x̂*_*i*−1_ and *x̂*_*i*_ measured about *ẑ*_*i*−1_.

**Fig 1:**
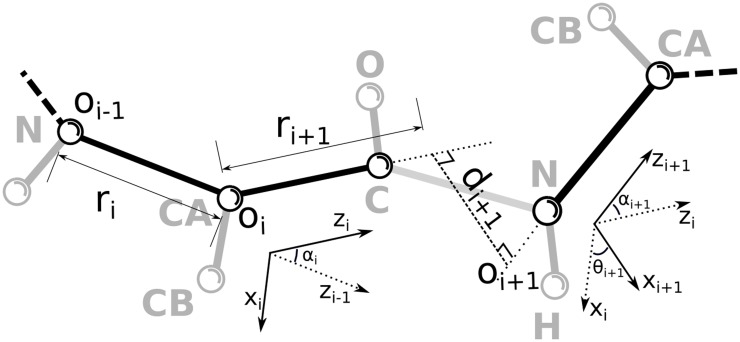
General graphical representation of a chain according to the Denavit-Hartenberg convention, as discussed in the text. Thick lines represent the physical bonds and spheres the atoms. *α*, *θ*, *r* and *d* are the DH parameters describing the chain and *o* is the origin of each local frame 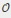. The structure of a portion of a protein backbone chain with all its heavy atoms is shown superimposed.

Fig. 1 shows how the DH parameters are defined for the general disconnected case (i.e. link offset *d*_*i*_ > 0). This is actually a typical case, when the structure of a protein backbone chain with all its heavy atoms is considered, since the *ω* torsional angle around the peptide bond is a hard degree of freedom with a well defined typical value. If *ω* is then kept strictly fixed, the DH convention allows to “spare” that degree of freedom, defining a disconnected chain as shown in Fig. 1. In the simplest case, when all bonds included in the DH description are connected with each other (i.e. all link offsets *d*_*i*_ = 0), the DH variables have a well defined physical meaning. Link lengths are bond lengths, link twists are supplementary of bond angles, and joint angles are torsional angles. The DH formalism is in this case equivalent to the one routinely used by software programs that reconstruct biomolecular structures subject to experimental restraints [18, 19] and that employ efficient internal dynamics algorithms that update only the values of torsional angles [20].

By using the DH definitions the matrix ${\text{R}}_{j}^{j-1}$ and the vector ${\vec{S}}_{j}^{j-1}$ can be explicitly expressed as

$$\begin{array}{c} R_{j}^{j-1}=\left(\begin{array}{ccc} \cos(\theta_{j}) & -\sin\left(\theta_{j}\right)\cos\left(\alpha_{j}\right) & \sin\left(\theta_{j}\right)\sin\left(\alpha_{j}\right)\\ \sin(\theta_{j}) & \cos\left(\theta_{j}\right)\cos\left(\alpha_{j}\right) & -\cos\left(\theta_{j}\right)\sin\left(\alpha_{j}\right)\\ 0 & \sin(\alpha_{j}) & \cos(\alpha_{j}) \end{array}\right) \end{array}$$ (2a)

$$\begin{array}{c} {\vec{S}}_{j}^{j-1}={\left(d_{j}\cos\left(\theta_{j}\right),\;d_{j}\sin\left(\theta_{j}\right),\;r_{j}\right)}^{T}. \end{array}$$ (2b)

With a more compact notation we rewrite Eq. (1) with the following

$$\begin{array}{c} a^{j-1}=T_{j}^{j-1}a^{j}, \end{array}$$ (3)

where ${\text{T}}_{j}^{j-1}$ is a 4 × 4 matrix given by

$$\begin{array}{c} T_{j}^{j-1}=\left(\begin{array}{cc} R_{j}^{j-1} & {\vec{S}}_{j}^{j-1}\\ 0 & 1 \end{array}\right) \end{array}$$ (4)

and **a** is the vector $a\;\text{=}\;{\text{(}\vec{a},\;1\text{)}}^{T}$. With this notation it is easier to relate any 𝒪_*j*_ with any other 𝒪_*i*_ (*j* > *i*); indeed the following equation holds:

$$\begin{array}{c} a^{i}=T_{j}^{i}a^{j}=T_{i+1}^{i}T_{i+2}^{i+1}\;\cdots \;T_{j}^{j-1}a^{j}. \end{array}$$ (5)

It should be now clear that the chain can be described by the whole set {*r*_*i*_, *α*_*i*_, *d*_*i*_, *θ*_*i*_}_*i* = 1,⋯,*N*_. For simplicity we denote {*r*_*i*_}_*i* = 1,⋯,*N*_ with **r**, {*α*_*i*_}_*i* = 1,⋯,*N*_ with *α*, {*d*_*i*_}_*i* = 1,⋯,*N*_ with **d** and {*θ*_*i*_}_*i* = 1,⋯,*N*_ with *θ*; the chain configuration is then given by the set {**r**, *α*, **d**, *θ*}.

### Performing the concerted local move

It is possible to deform an initial configuration {**r**_0_, *α*_0_, **d**_0_, *θ*_0_} by changing at least one of the parameters describing it.

**Fig 2:**
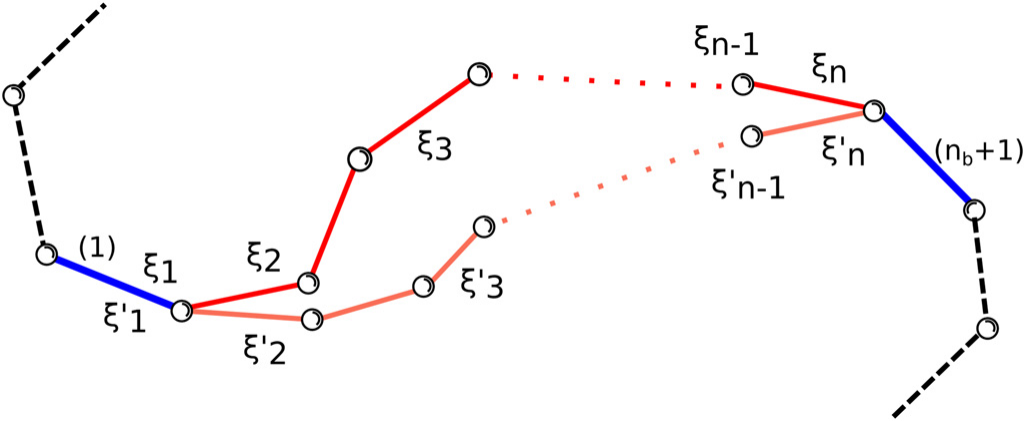
Schematic representation of the portion of a linear chain involved in a local modification. Bonds (1) and (*n*_*b*_ + 1) are colored in blue while all the others are in red. The degrees of freedom which are varied (*ξ*_1_, …, *ξ*_*n*_) are arbitrarily distributed inside the region. When they are concertedly changed to new values $\left(\xi_{1}^{\prime },\ldots ,\xi_{n}^{\prime }\right)$, the new pink configuration is obtained while all the bonds outside the region (in black) remain fixed in space.

Consider *n* DH parameters *ξ̃*_*μ*_, with *μ* = 1, ⋯, *n* and the *n*_*b*_ bonds to which the *n* parameters are related. The number of bonds to be considered is always smaller or equal to the number of DH parameters because in principle two or more parameters could be related to the same bond. As already stated we consider the general case in which these bonds can be non-consecutive. There are two particularly interesting bonds among the *n*_*b*_: the first and the last *i.e*. the one with the lowest label and the one with the highest one. These two bonds delimit the region of the chain we are interested in modifying with an opportune change of $\tilde{\xi }$, leaving the atoms outside this region unmodified (see Fig. 2). Such a change can be highly non-trivial and could not always be obtained: we will see later a condition that ensures us that this change can be performed.

For convenience, we re-label the bonds we are interested in with numbers from 1 to *n*_*b*_ + 1, where the latter is the first bond that remains fixed subsequent to the moved portion of the chain. The condition that needs to be imposed in order to ensure the locality of the change {**r**_0_, *α*_0_, **d**_0_, *θ*_0_} → {**r**, *α*, **d**, *θ*} is

$$\begin{array}{c} T_{n_{b}+1}^{1}\left(r,\;\alpha ,\;d,\;\theta \right)=T_{n_{b}+1}^{1}\left(r_{0},\;\alpha_{0},\;d_{0},\;\theta_{0}\right), \end{array}$$ (6)

that is requiring that the local reference frame 𝒪_*n*_*b*_+1_ does not move with respect to the first one. In order to explicit the variables $\tilde{\xi }$ inside the relation in Eq. (6) we define, with abuse of notation,

$$\begin{array}{c} T_{n_{b}+1}^{1}\left(\tilde{\xi }\right)\equiv T_{n_{b}+1}^{1}\left(r,\;\alpha ,\;d,\;\theta \right)-T_{n_{b}+1}^{1}\left(r_{0},\;\alpha_{0},\;d_{0},\;\theta_{0}\right) \end{array}$$ (7)

in such a way that Eq. (6) can be rewritten as

$$\begin{array}{c} T_{n_{b}+1}^{1}\left(\tilde{\xi }\right)=0. \end{array}$$ (8)

Given the form of ${\text{T}}_{i}^{j}$ (described in Eq. (4)), the 16 equations that are implicit in Eq. (8) can be reduced to 6 equations in the *n* variables $\tilde{\xi }$. Three equations are needed in order to set the translational part of ${\text{T}}_{i}^{j}$ and other three for the rotational part. We choose, for instance,

$$\begin{array}{c} \left\{\begin{array}{c} f_{1}\left(\tilde{\xi }\right)\equiv {\left[T_{n_{b}+1}^{1}\left(\tilde{\xi }\right)\right]}_{01}\\ f_{2}\left(\tilde{\xi }\right)\equiv {\left[T_{n_{b}+1}^{1}\left(\tilde{\xi }\right)\right]}_{02}\\ f_{3}\left(\tilde{\xi }\right)\equiv {\left[T_{n_{b}+1}^{1}\left(\tilde{\xi }\right)\right]}_{12}\\ f_{4}\left(\tilde{\xi }\right)\equiv {\left[T_{n_{b}+1}^{1}\left(\tilde{\xi }\right)\right]}_{03}\\ f_{5}\left(\tilde{\xi }\right)\equiv {\left[T_{n_{b}+1}^{1}\left(\tilde{\xi }\right)\right]}_{13}\\ f_{6}\left(\tilde{\xi }\right)\equiv {\left[T_{n_{b}+1}^{1}\left(\tilde{\xi }\right)\right]}_{23} \end{array}\right. \end{array}$$ (9)

or, in a more compact form, $\text{f}(\tilde{\xi })=\text{0}$. If *n* is greater than 6 and if the system of equation described in Eq. (9) is non degenerate then the solutions lie on a manifold with dimension *n* − 6. If, on the contrary, the system is degenerate the solutions lie on a manifold with dimension greater than *n* − 6.

Before to proceed it is important to notice that since the degrees of freedom $\tilde{\xi }$ can describe both spatial or angular quantities the space defined by these variables is in general dimensionally non-homogeneous. For this reason we introduce a set of *n* scalar multipliers *λ*_*i*_ chosen in such a way that every *ξ*_*i*_ = *λ*_*i*_ *ξ̃*_*i*_ is dimensionless. The space defined by the rescaled variables *ξ*_*i*_ is now homogeneous and an appropriate metric can be defined in it by means of the usual scalar product. In the case that a set of homogeneous variables are chosen as system variables (*e.g*. all dihedral angles) the introduction of the scalar multipliers is unnecessary but nonetheless it turns out to be useful (see Section “Tuning of fluctuations by rescaling variables”).

The problem of finding a new configuration *ξ* starting from an existing *ξ*_0_ in such a way that Eq. (8) is satisfied can now be visualized as the problem of *moving* on an (*n* − 6)-dimensional manifold embedded in an *n*-dimensional space. The most intuitive way to perform this non-trivial task is that of generating an intermediate configuration *ξ* ^′^ that may not lie on the manifold but that it is not far from it. This first step is called by other authors *pre-rotation* [15, 21], and we will adapt to this nomenclature. Starting from *ξ* ^′^ it is then possible to compute a true solution with numerical methods, *e.g*. root finding algorithms, or analytical ones [12].

In order to simplify the description of the pre-rotation step we introduce two new quantities *n*_*m*_ and *n*_*v*_ that are respectively the dimension of the tangent space *M* to the manifold at *ξ*_**0**_ and the dimension of the orthogonal space *V* to the manifold at *ξ*_**0**_. Obviously *n*_*m*_ + *n*_*v*_ = *n* and *n*_*v*_ = 6 if the system Eq. (9) is non-degenerate. In general *n*_*v*_ is the number of linearly independent functions in (9). The procedure we use to find a basis of the tangent space to the manifold takes advantage of the implicit function theorem in order to compute the derivatives $\frac{\partial \xi_{i}}{\partial \xi_{j}}$. Indeed we consider *n*_*m*_ among the *n* variables as independent and we denote them with the subscript *x*. The other *n*_*v*_ are labeled with a subscript *y* and will be written as a function of *ξ*_**x**_. With this notation we can write a set of *n*_*m*_ *n*-dimensional vectors that span the tangent space as

$$\begin{array}{c} e_{x,i}=\left(\frac{\partial \xi }{\partial \xi_{x,i}}\right), \end{array}$$ (10)

where the derivative $\left(\frac{\partial \xi }{\partial \xi_{x,i}}\right)$ can be performed by computing separately the contribute of the dependent variables *ξ*_*y*_ and that of the independent ones *ξ*_*x*_. The former (in the form of an *n*_*v*_-dimensional vector $\frac{\partial \xi_{y}}{\partial \xi_{x,i}}$) can be easily computed by applying the implicit function theorem

$$\begin{array}{c} \frac{\partial \xi_{y}}{\partial \xi_{x,i}}=-{\left(\frac{\partial f({\tilde{\xi }}_{0})}{\partial \xi_{y}}\right)}^{-1}\cdot \frac{\partial f({\tilde{\xi }}_{0})}{\partial \xi_{x,i}} \end{array}$$ (11)

while the latter is given by the *n*_*m*_ relations $\frac{{\partial \xi }_{x,j}}{\partial \xi_{x,i}}=\delta_{i,j}$. In the cases in which the matrix $\left(\frac{\partial f({\tilde{\xi }}_{0})}{\partial \xi_{y}}\right)$ is not invertible it is sufficient to choose a different set of *ξ*_**x**_ as independent variables. Vectors *e*_*x*,*i*_ can be orthonormalized to compute a basis {*ê*_*x*,*i*_}_*i*_ for the tangent space. The intermediate configuration *ξ* ^′^ can finally be computed by simply summing an arbitrary linear combination of *ê*_*x*,*i*_ to the initial configuration *ξ*_**0**_.

**Fig 3:**
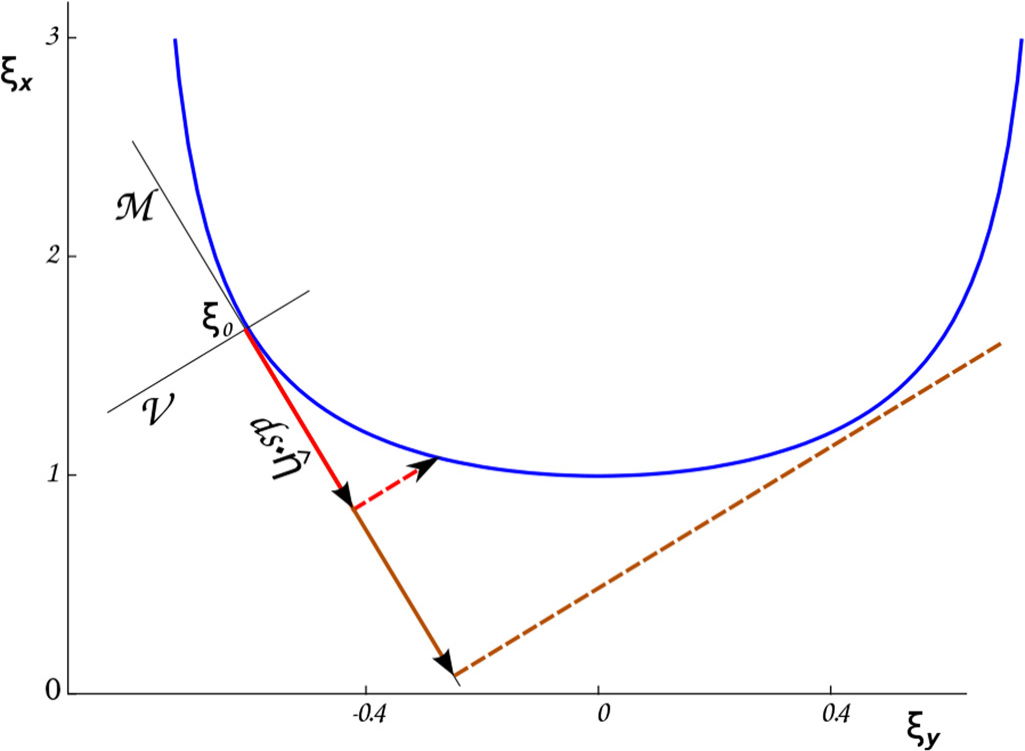
Graphical sketch of the updating of the conformations in the *n*-dimensional space of the degrees of freedom which are changed. The blue line represents the manifold of the solutions of Eq. (23), as discussed in the Section “Workout example” within “Material and Methods”. The manifold is plotted within the (*θ*, *ρ*) plane, with the independent variable being *ξ*_*x*_ = *ρ* and the dependent variable *ξ*_*y*_ = *θ*. *M* and *V* are respectively the tangent and the orthogonal space to the manifold in the starting conformation *ξ*_0_. The degrees of freedom are first changed along *M* (red continuous arrow) and a new conformation satisfying Eq. (9) is reached by moving along *V* (dashed red arrow). If the pre-rotation step along *M* is too large (brown continuous arrow), the post-rotation closure step along *V* (dashed brown arrow) fails to fall back to the manifold.

The intermediate configuration is an *open configuration* in which the position and orientation of the last reference frame do not correspond to the target ones. We therefore adjust the coordinates on the orthogonal space *V* by using a root-finding algorithm to obtain the final configuration. Fig. 3 depicts an example move in case the manifold is one-dimensional (the full example is addressed in the Section “Workout example”). The solution manifold (blue) and the initial configuration are represented. The pre-rotation step corresponds to the first update (thick red arrow) while the second step along the dotted line allows to converge back to the manifold by means of a root-finding algorithm. This is in general possible only for small enough pre-rotation steps. The brown arrows show the case when a too big pre-rotation step does not allow the procedure to converge back to the manifold.

A basis for *V* can be efficiently computed starting from the knowledge of a basis for *M* and by using *ad-hoc* algorithms (*e.g*. QR-decomposition algorithm). With this strategy the chain closure step usually takes few iterations of the Broyden’s root finding algorithm. Notice that it is in principle possible to use a root-finding algorithm that takes advantage of the easy-to-compute gradient $\frac{\partial f(\xi )}{\partial \xi }$ to boost the search for the solution. However our tests have shown that the time saved by the root-finding algorithm usually does not compensate for the time necessary for computing the gradient.

The full algorithm can be summarized in the following steps:

1. Compute a basis for the tangent space *M* at *ξ*_**0**_, as well as a basis for the orthogonal space *V*;
2. Choose an arbitrary direction $\hat{\eta }$ in *M* and an arbitrary step length *ds*;
3. Generate an intermediate configuration $\xi^{\prime }=\xi_{0}+ds\cdot \hat{\eta }$;
4. Use a root-finding algorithm in order to converge to a solution of Eq. (9) by moving on *V*;

### Detailed balance

In this section we will denote the three-dimensional configuration of an *N*-atom chain at time *t* with the 3*N*-dimensional vector $R^{t}=\{r_{1}^{t},r_{2}^{t},\cdots r_{N}^{t}\}$, where each $r_{k}^{t}={\left(x_{k}^{t},y_{k}^{t},z_{k}^{t}\right)}^{T}$ represents the three-dimensional position of atom *k* at time *t*.

In order to demonstrate that the scheme we proposed for changing the backbone configuration satisfies the detailed balance condition

$$\begin{array}{c} P\left(R^{t}\right)\Pi \left(R^{t}\to R^{t+\Delta t}\right)=P\left(R^{t+\Delta t}\right)\Pi \left(R^{t+\Delta t}\to R^{t}\right\} \end{array}$$ (12)

we will show separately that (1) with an appropriate change of variables, it is possible to uniformly sample the whole configurational space of the system without changes to the algorithm and (2) the probability of moving from *R* ^*t*^ to *R* ^*t*+Δ*t*^ can be chosen in such a way that Π(*R* ^*t*^ → *R* ^*t*+Δ*t*^) = Π(*R* ^*t*+Δ*t*^ → *R* ^*t*^).

We start by showing that point (1) is true. If the DH’s variables *α*, *θ*, **r** are used for describing the chain, the volume element in the configurational space

$$\begin{array}{c} dV^{t}=\prod_{k=1}^{N}dV_{k}^{t}=\prod_{k=1}^{N}dx_{k}^{t}dy_{k}^{t}dz_{k}^{t} \end{array}$$ (13)

can be rewritten as

$$\begin{array}{c} dV^{t}=\prod_{k=1}^{N}J_{k}^{t}d\alpha_{k}^{t}d\theta_{k}^{t}dr_{k}^{t}, \end{array}$$ (14)

where $J_{k}^{t}=\text{sin}(\alpha_{k}^{t}){\left(r_{k}^{t}\right)}^{2}$. Since the determinant $J^{s}=\prod_{k=1}^{N}J_{k}^{s}$ of the change of variables *R*_*t*_ → {*α*, *θ*, **r**} is not constant, a uniform sampling of the space of the DH’s variables does not result in a uniform sampling of the configurational space. In this case uniformity can be achieved by accepting the configuration change {*α*, *θ*, **r**}_1_ → {*α*, *θ*, **r**}_2_ with probability $p=\text{min}\left(1,\frac{J^{t+\Delta t}}{J^{t}}\right)$. Such essential choice compromises efficiency both by increasing the number of calculations per time step and by reducing the probability of obtaining a new configuration. With our approach the problem is overcome without performing any additional time-consuming calculation. Indeed it is easy to notice that the algorithm proposed in the previous section does not rely on the particular form of *ξ* or $\text{f}(\tilde{\xi })$ but rather on the possibility of computing $f(\tilde{\xi })$ and its derivatives. For this reason every differentiable, invertible function of $\tilde{\xi }$, whose inverse is differentiable, can be used as degree of freedom of the system without jeopardizing the efficiency of the full scheme. If, for instance, $\psi =g(\tilde{\xi })$ is used as degree of freedom, the following trivial relations holds:

$$\begin{array}{c} f\left(\psi \right)=f\left(g\left(\tilde{\xi }\right)\right) \end{array}$$ (15a)

$$\begin{array}{c} \frac{\partial f(\psi )}{\partial \tilde{\xi }}=\frac{\partial f(\psi )}{\partial \psi }\frac{\partial g(\tilde{\xi )}}{\partial \tilde{\xi }}. \end{array}$$ (15b)

If DH’s parameters can be interpreted as bond lengths, bond angles and torsional angles (*θ* is always a torsional angle, *α* is a bond angle for bonds connected to the previous one, *r* is a bond length for bonds connected to both the previous and the subsequent one) it is sufficient to use the variables ${\tilde{\xi }}_{\alpha }=cos(\alpha )$ and ${\tilde{\xi }}_{r}=\frac{r^{3}}{3}$ as new degrees of freedom in order to guarantee the determinant of the Jacobian of the change of variables to be constant: this implies that the new configuration can always be accepted. This result relies only on the fact that the Jacobian does not depend on *ξ*; we derived it independently from the values of the rescaling factors.

Notice that even if the inverse of the cosine function is not differentiable in 0 and *π*, these points are at the boundary of the domain in which the bond angle *α* is defined. Hence the analysis is valid in the open domain (0, *π*) but not in its closure. The same is true for $\frac{r^{3}}{3}$, whose inverse is not differentiable in *r* = 0. Of course these drawbacks are not relevant for most applications, since *α* = 0, *π* and *r* = 0 are far from physical values. Therefore a uniform sampling of the space of the new variables $\left\{{\tilde{\xi }}_{\alpha },{\tilde{\xi }}_{r},\theta \right\}$ is sufficient to ensure a uniform sampling of the configurational space.

In order to ensure the detailed balance condition to be valid it is now necessary to check whether the step size *ds* that has been used in the forward update *ξ*_1_ → *ξ*_2_ is the same that would be necessary for the reverse transformation *ξ*_2_ → *ξ*_1_. Indeed the step size is typically chosen from a fixed probability distribution and therefore if the backward step size *ds* ^′^ is different from the forward one, it is necessary to take into account the different probability of proposing such a step. The backward step can be easily computed as the norm of the projection of *ξ*_2_ − *ξ*_1_ onto the tangent space to the manifold at *ξ*_2_. If the step size is chosen from a normal distribution with zero average and variance *σ* ^2^ it is sufficient to accept the update with probability

$$\begin{array}{c} P=\min\left(1,\exp\left(\frac{ds^{2}-ds^{\prime 2}}{2\sigma^{2}}\right)\right) \end{array}$$ (16)

to ensure the equivalence between forward and backward probability. In many practical cases $\frac{ds^{\prime }}{ds}$ is close to one and, as a consequence, the probability of rejecting a Monte Carlo move is negligible. This calculation can be done for whatever choice of the factors *λ*_*i*_. The detailed balance can therefore be satisfied independently from the choice of each *λ*_*i*_.

### Tuning fluctuations by rescaling variables

The role of rescaling factors *λ*_*i*_ previously introduced can be further exploited. As already briefly discussed, their introduction has been necessary to map the *original* non-homogeneous space of the variables $\tilde{\xi }$ in the homogeneous one of the variables $\xi =\lambda \cdot \tilde{\xi }$ where a metric can be defined. But on top of that they can be used in order to tune the fluctuations of the corresponding degrees of freedom.

Given a starting configuration ${\tilde{\xi }}_{0}$ suppose that some of the ${\tilde{\xi }}_{0,i}$ are hard degrees of freedom (${\tilde{\xi }}_{0,H}$) while the others are soft (${\tilde{\xi }}_{0,S}$). We can use two different values of *λ*_*i*_, depending on the class of the corresponding degrees of freedom, thus mapping

$$\begin{array}{c} {\tilde{\xi }}_{0}={\left({\tilde{\xi }}_{0,S},{\tilde{\xi }}_{0,H}\right)}^{T}\to {\left(\lambda_{S}\cdot {\tilde{\xi }}_{0,S},\;\lambda_{H}\cdot {\tilde{\xi }}_{0,H}\right)}^{T}=\xi_{0}, \end{array}$$ (17)

where *λ*_*H*_ > *λ*_*S*_. As described in previous sections the algorithm is applied to the initial conformation in the homogeneous, deformed space. For simplicity we consider in the following discussion the particular case in which the soft degrees of freedom correspond to the independent variables, in such a way that the new configuration *ξ* can be written as a function of the variation vectors Δ*ξ*_*S*_ and Δ*ξ*_*H*_ = ∇_*ξ*_*S*__ *ξ*_*H*_ ⋅ Δ*ξ*_*S*_ as

$$\begin{array}{c} \xi =\xi_{0}+\Delta \xi ={\left(\lambda_{S}\cdot {\tilde{\xi }}_{0,S}+\Delta \xi_{S},\;\lambda_{H}\cdot {\tilde{\xi }}_{0,H}+\Delta \xi_{H}\right)}^{T}, \end{array}$$ (18)

where ∇_*ξ*_*S*__ *ξ*_*H*_ is the matrix of the derivatives of the hard degrees of freedom with respect to the soft ones.

It is possible to express the norm of the variation vector for independent variables $\Delta {\tilde{\xi }}_{S}$ as a function of the size of the actual move performed in our algorithm, the step size *ds* in the tangent space: $ds={\left(\;\lambda_{S}^{2}||\Delta {\tilde{\xi }}_{S}|{|}^{2}+\lambda_{H}^{2}||\Delta {\tilde{\xi }}_{H}|{|}^{2}\;\right)}^{\frac{1}{2}}$. The previous equation allows to define the function $g_{\lambda_{s},\lambda_{H}}\left({\tilde{\xi }}_{S},{\tilde{\xi }}_{H}\right)$ through $||\Delta {\tilde{\xi }}_{S}||=\frac{g_{\lambda_{s},\lambda_{H}}\left({\tilde{\xi }}_{S},{\tilde{\xi }}_{H}\right)}{\lambda_{S}}\;ds$. The function $g_{\lambda_{s},\lambda_{H}}\left({\tilde{\xi }}_{S},{\tilde{\xi }}_{H}\right)$ describes the reduction of the variation of the soft independent variables, for a fixed step size *ds*, due to the orientation of the tangent space with respect to the space of independent variables. It depends, in general, both on the position of the initial point in the manifold and on the chosen rescaling factors.

Each point of the deformed manifold can be remapped to the original manifold with the inverse transformation $\tilde{\xi }=\frac{1}{\lambda_{i}}\xi$. Therefore the new configuration found in Eq. (18) corresponds to a final configuration in the original space equal to

$$\begin{array}{c} \tilde{\xi }={\left({\tilde{\xi }}_{0,S}+\frac{1}{\lambda_{S}}\left[g_{\lambda_{s},\lambda_{H}}\left({\tilde{\xi }}_{S},{\tilde{\xi }}_{H}\right)\;ds\right]\;{\hat{\Delta \tilde{\xi }}}_{S},\;{\tilde{\xi }}_{0,H}+\frac{1}{\lambda_{H}}\left[g_{\lambda_{s},\lambda_{H}}\left({\tilde{\xi }}_{S},{\tilde{\xi }}_{H}\right)\;ds\right]\;\nabla_{\xi_{S}}\xi_{H}\cdot {\hat{\Delta \tilde{\xi }}}_{S}\right)}^{T}, \end{array}$$ (19)

where ${\hat{\Delta \tilde{\xi }}}_{S}$ is a normalized vector.

The previous equation holds also in the *λ*_*S*_ = *λ*_*H*_ = 1 case, when no rescaling occurs. The rescaling factor *K*_*S*_ for the variation of the independent variables ${\tilde{\xi }}_{S}$ as a consequence of the introduction of *λ*_*S*_, *λ*_*H*_ ≠ 1 is then easily computed:

$$\begin{array}{c} K_{S}\left({\tilde{\xi }}_{S},{\tilde{\xi }}_{H}\right)=\frac{1}{\lambda_{S}}\frac{g_{\lambda_{s},\lambda_{H}}\left({\tilde{\xi }}_{S},{\tilde{\xi }}_{H}\right)}{g_{1,1}\left({\tilde{\xi }}_{S},{\tilde{\xi }}_{H}\right)} \end{array}$$ (20)

while the rescaling factor *K*_*H*_ for the variation of the hard degrees of freedom ${\tilde{\text{ξ}}}_{H}$ reads

$$\begin{array}{c} K_{H}\left({\tilde{\xi }}_{S},{\tilde{\xi }}_{H}\right)=\frac{\lambda_{S}}{\lambda_{H}}\;K_{S}\left({\tilde{\xi }}_{S},{\tilde{\xi }}_{H}\right)\frac{||\nabla_{\xi_{S}}\xi_{H}\cdot {\hat{\Delta \tilde{\xi }}}_{S}||}{||\nabla_{{\tilde{\xi }}_{S}}{\tilde{\xi }}_{H}\cdot {\hat{\Delta \tilde{\xi }}}_{S}||}. \end{array}$$ (21)

This is the central equation of this section, as it shows to what extent the variation vector for hard degrees of freedom is modified differently than for soft degrees of freedom, due to the rescaling of the original variables. Besides the global tuning factor $\frac{\lambda_{S}}{\lambda_{H}}$ a second factor $\frac{||\nabla_{\xi_{S}}\xi_{H}\cdot {\hat{\Delta \tilde{\xi }}}_{S}||}{||\nabla_{{\tilde{\xi }}_{S}}{\tilde{\xi }}_{H}\cdot {\hat{\Delta \tilde{\xi }}}_{S}||}$ appears, related to the local geometrical properties of the considered manifold.

For one-dimensional manifolds, it is easy to see that $\nabla_{\xi_{S}}\xi_{H}=\frac{\lambda_{H}}{\lambda_{S}}\nabla_{{\tilde{\xi }}_{S}}{\tilde{\xi }}_{H}$, so that $K_{S}({\tilde{\xi }}_{S},{\tilde{\xi }}_{H})=K_{H}({\tilde{\xi }}_{S},{\tilde{\xi }}_{H})$; the variations of both the hard and the soft degrees of freedom are rescaled in the same way, as if a new effective step size $ds({\tilde{\xi }}_{S},{\tilde{\xi }}_{H})=K_{S}({\tilde{\xi }}_{S},{\tilde{\xi }}_{H})\;ds$ were used.

For manifold of higher dimension, if $\nabla_{\xi_{S}}\xi_{H}\neq \frac{\lambda_{H}}{\lambda_{S}}\nabla_{{\tilde{\xi }}_{S}}{\tilde{\xi }}_{H}$ then $K_{S}({\tilde{\xi }}_{S},{\tilde{\xi }}_{H})\neq K_{H}({\tilde{\xi }}_{S},{\tilde{\xi }}_{H})$ and the *λ* factors can effectively be used in order to tune the amplitude of the fluctuations of hard degrees of freedom with respect to the soft ones. It is obviously not easy to quantify ‘a priori’ the local geometric factor $\frac{||\nabla_{\xi_{S}}\xi_{H}\cdot {\hat{\Delta \tilde{\xi }}}_{S}||}{||\nabla_{{\tilde{\xi }}_{S}}{\tilde{\xi }}_{H}\cdot {\hat{\Delta \tilde{\xi }}}_{S}||}$.

In the most general case, with no restriction on the soft degrees of freedom being either independent or dependent variables, we expect to find qualitatively similar results both for high-dimensional and one-dimensional manifolds.

### Workout example

In order to better explain each step of the algorithm we provide a simple example that can be solved exactly. Beyond the context of concerted local movements in chain molecules, the simplest possible case for our algorithm corresponds to the motion along a one-dimensional manifold defined by a single constraint within a two-dimensional space. In this spirit, we consider a physical system in which a single particle is constrained to move within the (*x*, *y*) plane on the right branch (*x* ≥ 1) of a hyperbola of equation

$$\begin{array}{c} x^{2}-y^{2}-1=0. \end{array}$$ (22)

We introduce new polar coordinates $\left(\tilde{\rho }=\sqrt{x^{2}+y^{2}},\tilde{\theta }=\text{atan}\left(\frac{y}{x}\right)\right)$ that, in this example, will have the same role the DH parameters have for the description of a chain molecule. Notice that *θ̃* is defined in the open interval $\left(-\frac{\pi }{4},\frac{\pi }{4}\right)$. We also introduce two scalar quantities *λ*_*ρ*_ and *λ*_*θ*_ defined in such a way that *ρ* = *λ*_*ρ*_ *ρ̃* and *θ* = *λ*_*θ*_ *θ̃* are both dimensionless quantities. Constraints are defined by the analogous of Eq. (9)

$$\begin{array}{c} f_{1}\left(\tilde{\rho },\tilde{\theta }\right)\;:\;{\tilde{\rho }}^{2}\cos\left(2\tilde{\theta }\right)-1=0 \end{array}$$ (23)

that, as a function of the rescaled variables becomes

$$\begin{array}{c} f_{1}\left(\rho ,\theta \right)\;:\;\frac{\rho^{2}}{\lambda_{\rho }^{2}}\cos\left(2\frac{\theta }{\lambda_{\theta }}\right)-1=0. \end{array}$$ (24)

The tangent space *M* and the orthogonal space *V* in the initial configuration (*ρ*_0_, *θ*_0_) can be easily computed starting from the expression of the derivatives of *f*_1_(*ρ*, *θ*)

$$\begin{array}{c} \left\{\begin{array}{c} \frac{\partial f_{1}\left(\rho ,\theta \right)}{\partial \rho }=2\frac{\rho }{\lambda_{\rho }^{2}}\cos\left(2\frac{\theta }{\lambda_{\theta }}\right)\\ \frac{\partial f_{1}\left(\rho ,\theta \right)}{\partial \theta }=-2\frac{\rho^{2}}{\lambda_{\rho }^{2}\lambda_{\theta }}\sin\left(2\frac{\theta }{\lambda_{\theta }}\right) \end{array}\right. \end{array}$$ (25)

by means of the implicit function theorem. Indeed, if the initial configuration is such that $\frac{\partial f_{1}(\rho ,\theta )}{\partial \theta }\neq 0$ (*i.e*.*θ* ≠ 0) it is possible to choose *ρ* as independent variable and to compute the derivative $\frac{\partial \theta }{\partial \rho }=\frac{\lambda_{\theta }}{\rho }\text{cotan}\left(2\frac{\theta }{\lambda_{\theta }}\right)$. The tangent space *M* is therefore generated by the vector $\eta (\rho ,\theta )={\left(1,\frac{\partial \theta }{\partial \rho }\right)}^{T}$ and any intermediate configuration can be selected as $\left(\rho^{\prime },\theta^{\prime })=(\rho_{0},\theta_{0})+ds\cdot \hat{\eta }(\rho_{0},\theta_{0}\right)$, where *ds* is an arbitrary step-size and $\hat{\eta }$ is normalized. Once *η* is computed, the QR algorithm is used in order to generate a basis for the orthogonal space *V*. In this simple example *V* is one-dimensional and the generating vector can be computed explicitly as $\eta^{⊥}(\rho ,\theta )={\left(-\frac{\partial \theta }{\partial \rho },1\right)}^{T}$. A root finding algorithm is finally used in order to find a solution to the equation *f*_1_(*ρ*, *θ*) = 0 with $(\rho ,\theta )=(\rho^{\prime },\theta^{\prime })+k\cdot {\hat{\eta }}^{⊥}$ and with varying *k*. In case *θ* = 0, it is not possible to use *ρ* as the independent variable, and *θ* has to be chosen instead. Notice that in order to ensure numerical stability it is safer to use *θ* as independent variable in a suitable interval centered in *θ* = 0. Also, the existence of a solution is in general ensured only for small enough step sizes, as depicted in Fig. 3.

We now consider within this example the effect of introducing the rescaling factors *λ*_*ρ*_ and *λ*_*θ*_, as discussed in the previous section. We assume *θ̃* ≠ 0, so that *ρ* can be used as the independent variable. In this case we see that

$$\begin{array}{c} \frac{\partial \theta }{\partial \rho }=\frac{\lambda_{\theta }}{\lambda_{\rho }}\frac{\partial \tilde{\theta }}{\partial \tilde{\rho }} \end{array}$$ (26)

and therefore any rescaling induced for *dρ* is applied to *dθ* as well, as expected for a one-dimensional manifold. The relation between the step size *ds* tangent to the manifold and the variation *dρ̃* of the independent unrescaled variable is $ds=d\tilde{\rho }{\left[\lambda_{\rho }^{2}+\lambda_{\theta }^{2}{\left(\frac{\partial \tilde{\theta }}{\partial \tilde{\rho }}\right)}^{2}\right]}^{\frac{1}{2}}$, that allows to recover the function $g_{\lambda_{\rho },\lambda_{\theta }}\left(\tilde{\rho },\tilde{\theta }\right)=\frac{\lambda_{\rho }}{ds/d\tilde{\rho }}={\left[1+\frac{\lambda_{\theta }^{2}}{\lambda_{\rho }^{2}}\frac{{\text{cotan}}^{2}\left(2\tilde{\theta }\right)}{{\tilde{\rho }}^{2}}\right]}^{-\frac{1}{2}}$. We finally obtain the rescaling function from Eq. (20) as

$$\begin{array}{c} K\left(\tilde{\rho },\tilde{\theta }\right)=\frac{1}{\lambda_{\rho }}\frac{{\left[1+\frac{1}{{\tilde{\rho }}^{2}}{\mathrm{cotan}}^{2}\left(2\tilde{\theta }\right)\right]}^{\frac{1}{2}}}{{\left[1+\frac{\lambda_{\theta }^{2}}{\lambda_{\rho }^{2}{\tilde{\rho }}^{2}}{\mathrm{cotan}}^{2}\left(2\tilde{\theta }\right)\right]}^{\frac{1}{2}}}\;. \end{array}$$

## Results

In this section we present some applications of our method for the study of polypetide molecular systems, preceded by a test of detailed balance and of how the fluctuations of different degrees of freedom can be tuned by using rescaled variables. In polypeptide chains the rules of quantum chemistry constrain bond lengths and bond angles to fluctuate slightly around known values and double bonds to be approximately planar. The flexibility of the chain is therefore mainly due to the variation of *ϕ*, *ψ* Ramachandran’s angles. In order to mimic this behavior, hard degrees of freedom are typically strongly constrained by using stiff quadratic potentials. This is not necessary with our approach because the hard degrees of freedom can be kept frozen in their minimum energy value thus reducing the number of degrees of freedom.

For this reason, in all applications discussed below we consider only the Ramachandran’s *ϕ* and *ψ* torsional angles as degrees of freedom of the system; since *ω* is kept fixed, the set of bonds considered in the DH description is disconnected, as shown in Fig. 1. In practice, in our simulations the system Eq. (9) is always not degenerate, in such a way that the solution manifold has co-dimension 6.

### Detailed balance

**Fig 4:**
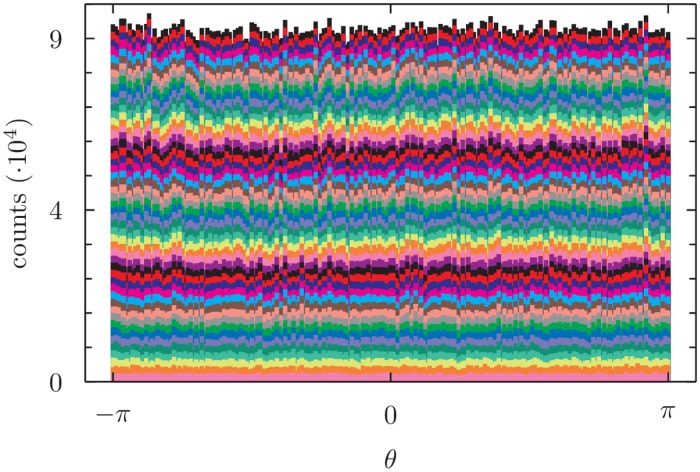
Distribution of the values of torsional angles of a 67 residues long protein (1CTF) during a 3.5 ⋅ 10^5^ time-steps Monte-Carlo simulation. Each of the stacked barchart is relative to a different torsional angle. At each simulation step the structure is deformed by applying our algorithm to a randomly chosen portion of the chain (with probability 0.7) or by a pivot move (with probability 0.3). In the former case the step size *ds* is chosen from a normal distribution with mean 0 *rad* and variance 0.08 *rad* ^2^. In the latter case a randomly chosen *ϕ* or *ψ* angle is perturbed by adding to it a quantity that is chosen from a normal distribution with mean 0 *rad* and variance 0.4 *rad* ^2^.

In order to verify that the detailed balance is satisfied we performed a Monte Carlo simulation on a 63-residue long protein fragment (pdb code 1CTF) by using our algorithm. We allowed only the Ramachandran’s *ϕ* and *ψ* angles to vary during the simulation; these modifications were obtained by randomly selecting either a Pivot move around a randomly selected bond or our locally concerted move on a set of randomly selected angles. The introduction of the Pivot move was necessary to move the last bond of the chain and thus ensure simulation ergodicity. Fig. 4 shows the distribution of the values of different torsional angles of a protein chain that has been simulated using the proposed schema. As expected when detailed balance holds, the distribution is flat, within natural stochastic fluctuations.

### Fluctuation tuning by rigidity rescaling

**Fig 5:**
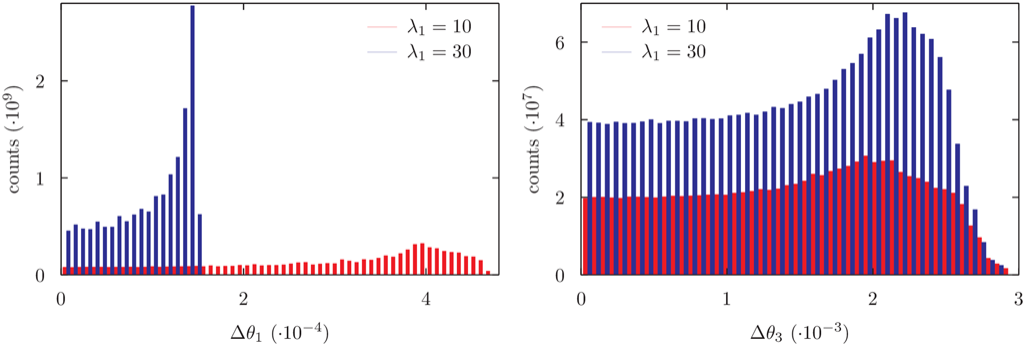
Distribution of variability of *θ*_1_ and *θ*_3_ angles for different values of *λ*_1_ for a 9 degrees of freedom chain. The distribution of *θ*_3_ is largely not affected by the change of *λ*_1_ while the distribution of *θ*_1_ is rescaled according to Eq. (19).

We also studied how the dynamics of the exploration of the solution manifold is affected by the rigidity factors *λ*_*i*_ used to rescale the original variables (see Materials and Methods). This has been done by comparing the distribution of the fluctuations Δ*θ* of two different torsional angles upon changing the rigidity of one of the two. The data have been obtained by performing short simulations with fixed step-size *ds* = 0.01 along a randomly chosen direction onto the tangent space to the manifold. First a nine degrees of freedom fragment, corresponding to a three-dimensional manifold, was explored for different values of *λ*_1_. Fig. 5 shows the distribution of the fluctuations of the corresponding torsional angle *θ*_1_ and of another torsional angle (*θ*_3_) used as negative control. A rescaling of *λ*_1_ by a factor of 3 does not globally affect the distribution of Δ*θ*_3_, but rescales the corresponding distribution of Δ*θ*_1_ by a factor roughly 3. This shows that the main role in Eq. (19) is played by the global rescaling factor 1/*λ*, wheres the role of local manifold geometry is in practice less relevant, at least in the considered example. On the other hand, the effect of local manifold geometry may explain some reshaping that can be appreciated in the plotted distributions apart from the global rescaling.

**Fig 6:**
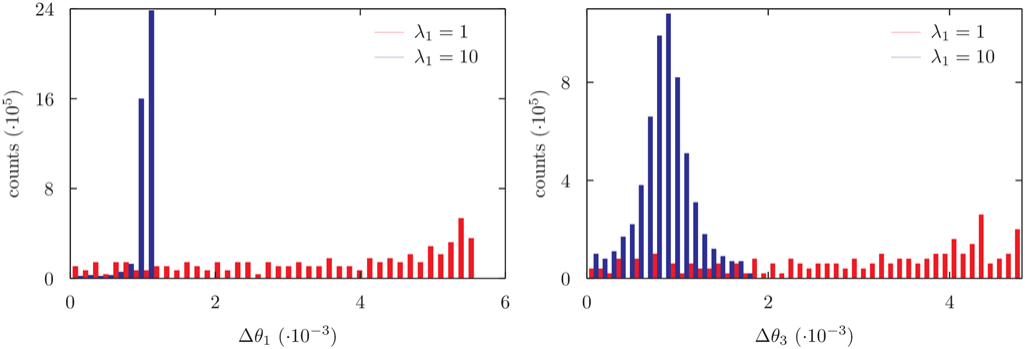
Distribution of variability of *θ*_1_ and *θ*_3_ angles for different values of *λ*_1_ for a 7 degrees of freedom chain. In this case it is not possible to change a single dof without altering the others: as a consequence a rescaling of *λ*_1_ affects the distributions of *θ*_1_ as well as of the other angles (shown: *θ*_3_). In this case the effect of changing the rigidity of one or more parameters is the same as rescaling the step size used during the simulation.

Similarly, Fig. 6 shows the distribution of the fluctuations of the angles *θ*_1_ and *θ*_3_ obtained while exploring the solution manifold of a seven degrees of freedom fragment with a fixed step-size *ds* for different values of *λ*_1_. In this case the solution manifold is one-dimensional and therefore the fluctuations of *θ*_1_ and *θ*_3_ are always related. Indeed, after the rigidity *λ*_1_ is increased, both distributions are globally rescaled by the same quantity. The dynamics on the deformed manifold corresponds, in this case, to a dynamics on the original manifold with a rescaled step size. For the one-dimensional example as well, some reshaping can be seen in the plotted distributions apart from the global rescaling. Again, this observation may be explained as an effect of local manifold geometry, consistently with Eq. (19).

### Exploring the conformational space

The first application we describe is the exploration of the whole conformational space of a small portion of a polypeptide, *i.e*. finding all configurations of that fragment that are compatible with the locality constraints. The problem can be resolved by finding a method to compute all the solutions of Eq. (9). This is not a simple task when the number of degrees of freedom is large but, on the contrary, if the number of variables is 7 the exploration of the manifold is simple. In this case the manifold has dimension 1 and so it is sufficient to move on the manifold always along the same direction. This can be done by choosing at each step *k* an initial direction ${\hat{\eta }}_{k}$ in such a way that ${\hat{\eta }}_{k-1}\cdot {\hat{\eta }}_{k}>0$. In practice, we choose ${\hat{\eta }}_{k}$ to be parallel to the projection of ${\hat{\eta }}_{k-1}$ onto the tangent space to the manifold in the actual configuration.

**Fig 7:**
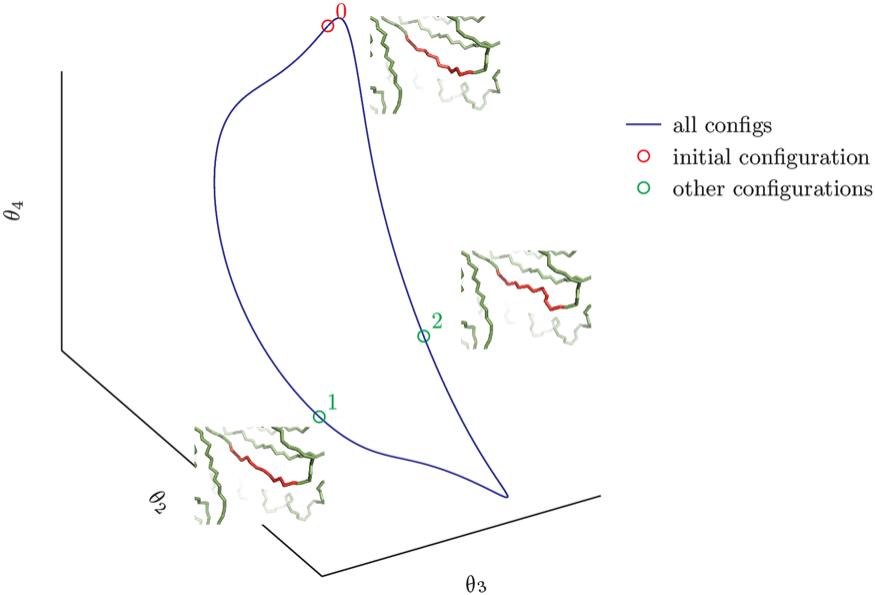
Three-dimensional projection of the solution manifold. The whole manifold lies in a seven-dimensional space. Some configurations are highlighted: the starting configuration is emphasized with a red circle while two other possible solutions are emphasized in green. The red part of the structure is the portion which has been modified with our moves.

Fig. 7 shows a three-dimensional projection of a one-dimensional manifold obtained by changing 7 consecutive *ϕ* and *ψ* Ramachandran’s angles of a portion of a protein. Some configurations of the polypeptide that have been generated during the exploration are also plotted. There are no other configurations of the selected region that are compatible with the constraints imposed by the locality requirement and that can be generated with a continuous modification of the original configuration.

**Fig 8:**
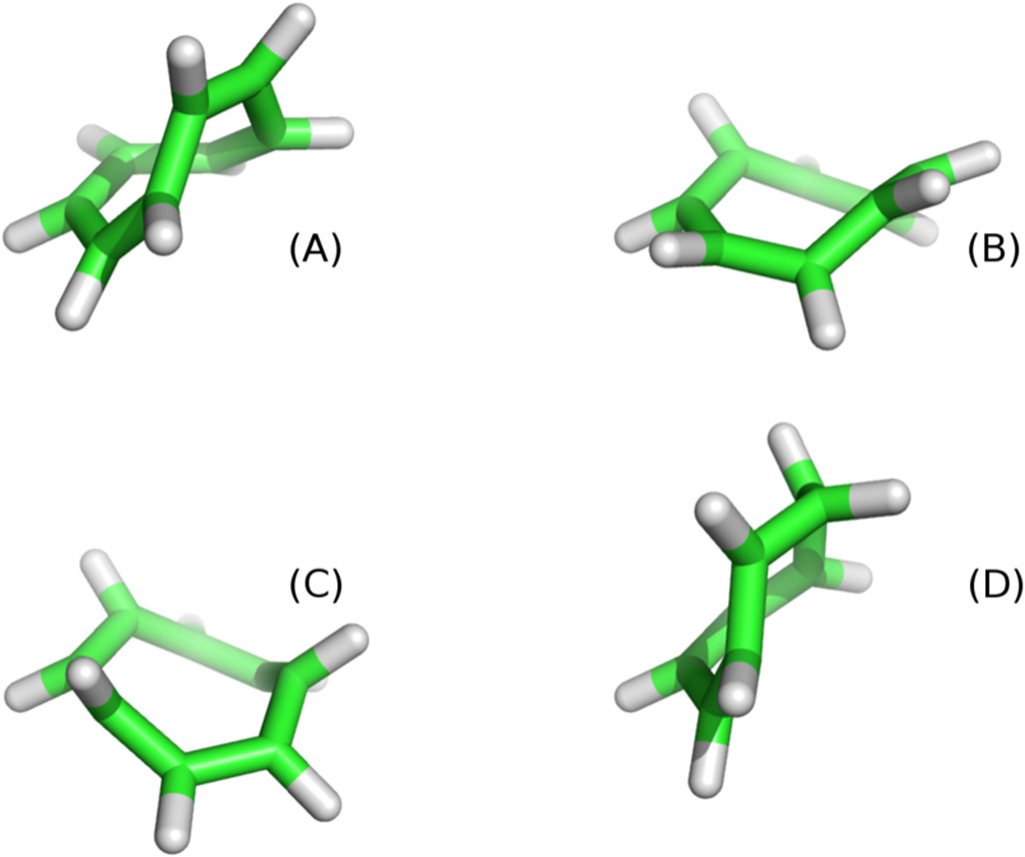
Four conformations of the ciclic molecule cyclooctatetraene obtained while exploring its whole conformational space by changing the 8 torsional angles degrees of freedom. Bond length and bond angles are kept fixed. This is a toy model to illustrate the efficiency of the method: the molecule alternates double and single bonds and therefore half of the torsional angles are constrained and only four are completely free. Images of molecular structures have been generated with PyMol [22].

**Fig 9:**
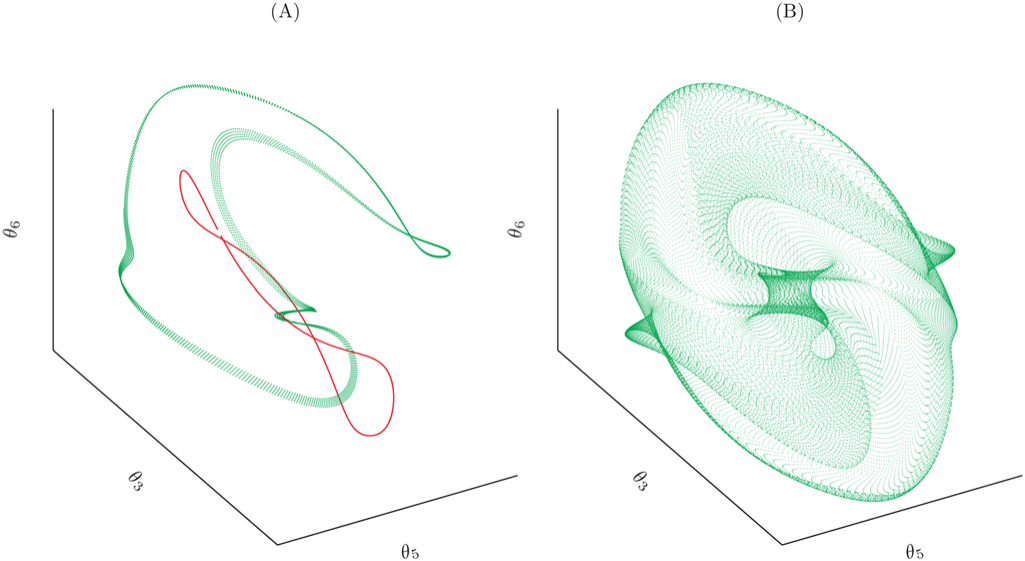
A three-dimensional projection of the solution manifold for our model of cyclooctotetraene molecule (B) and a schematics of how the manifold has been explored (A). First a set of seven angles are chosen and the relative one-dimensional manifold is visited (red line). Then the one-dimensional manifold relative to a different choice of degrees of freedom is explored using each of the generated structure as a starting point (green dots).

Higher dimensional manifolds can be explored as well but, in these cases, there is not a general strategy that allows an efficient exploration of the whole space. This exploration can be achieved with a Monte-Carlo simulation or with *ad hoc* procedure as in the next example. As a proof of concept, with the only purpose of showing the viability of the method in higher dimensions, we consider a small cyclic molecule (cyclooctatetraene) and we assume that all its 8 torsional angles are soft degrees of freedom that can be modified. Fig. 8 shows four conformations of cyclooctatetraene obtained with our procedure and Fig. 9 shows the whole bidimensional solution manifold.

We highlight the efficiency of the method. The full exploration of cyclooctatetraene configurational space has been performed in about 5 minutes on a single core 2.0 Ghz computer, collecting more than 10^5^ different structures with a sample-step of 10^−2^ radians.

### Protein backbone mobility

In this section we describe how it is possible to estimate the *mobility* of a portion of protein backbone (*local backbone mobility*) by using a simple schema based on the algorithm proposed in this paper. The hypothesis we use is that the local mobility is proportional to the number of configurations that can be explored locally without modifying the rest of the chain, the *local backbone volume*. In principle, the number of configurations taken into account in this counting could be reduced by eliminating those conformations that exhibit steric clashes. Also, it would be possible to introduce a pair-wise potential in order to consider interaction effects. Here we limit the study of the mobility to non-interacting chains.

Consider the 3*N*-dimensional configurational space describing the position of each atom composing the system. In analogy with the usual definition of entropy, we define the *local backbone entropy* as the logarithm of the local backbone volume, that is the volume of the solution manifold measured in the 3*N*-dimensional configurational space. This volume can be computed by integrating the Gramian of the transformation {*α*, *θ*, **r**} → *R* ^*t*^ on the solution manifold in the DH’s variable space. The Gramian function specifies how the (*n* − 6)-dimensional volume element of the manifold in the space of DH parameters is mapped into the 3*N*-dimensional configurational space. If **s** are the coordinates on the manifold, determined within an orthonormal system of basis vectors in the tangent space, the Jacobian of the transformation can be written as ∇_**s**_ *R* ^*t*^, and the Gramian is computed as

$$\begin{array}{c} G\left(s\right)={\left\{det\;\left[{\left(\nabla_{s}R^{t}\right)}^{T}\;\nabla_{s}R^{t}\right]\right\}}^{\frac{1}{2}}, \end{array}$$ (27)

(see [23, 24]).

Values of entropy can be assigned to different protein fragments, *i.e*. to different subsets of degrees of freedom, by considering the corresponding manifolds defined by their concerted variations. We first estimate the entropy *S*(*i*) associated to a single residue *i* as the sum of the entropies computed for all the different fragments comprising that residue. For simplicity, we restrict our calculation to fragments that comprise seven consecutive *ϕ* and *ψ* Ramachandran’s angles. We then call the map *i* ↦ *A* × exp(*S*(*i*)) the *mobility profile* of the structure. The factor *A* is a proportionality constant which has the dimension of an Angstrom squared.

**Fig 10:**
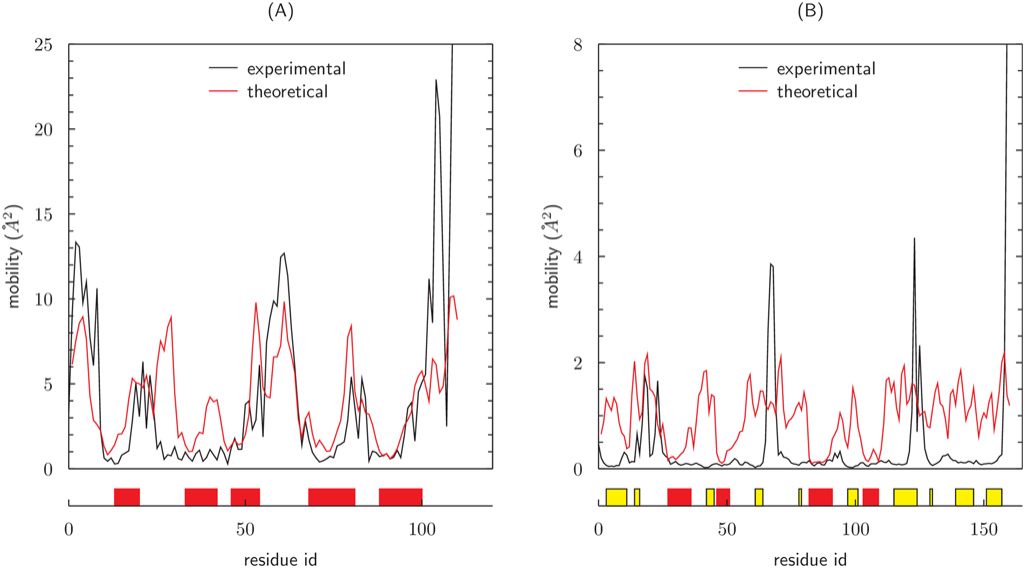
Comparison between the experimental mobility and the theoretical *mobility profile* of different structures. Data in (A) are relative to an all-*α* protein (1YGM) while (B) shows the mobility of mixed *β* and *α* protein (2ITH). The experimental profile has been computed as the variance in the position of *α*-carbons in different *NMR* models of the same protein. Red lines represent theoretical calculations, black lines experimental values. Boxes below the horizontal axis locate the position of secondary structures along the chain: red for *α*-helices and yellow for *β*-sheet. The matching between the theorethical and experimental profiles has been obtained by optimizing the proportionality constant *A* with a least square fit. In (A) the fit has been computed over the whole length of the chain, while in (B) the fit concerned only the *α* regions.

In Fig. 10, we compare the *mobility profile* with the corresponding experimental data, *i.e*. the variance of the positions of the *α* carbon atoms in different NMR models of the same structure. Note that the predicted mobility profile is matched to the experimental one by fitting *A*, so that only the ratio between the mobilities of different regions of the same protein structure can be considered as a real prediction of our model.

Remarkably, the mobility of 1YGM, an *all-*α* structure*, is described with a good degree of accuracy by the theoretical estimations. This fact, confirmed by similar analysis on other *all-*α* structures* (data not shown) suggests that the local geometrical constraints of the protein backbone taken into account by our method are enough to predict the relative mobility of helical and non helical regions. The surprising conclusion is that the presence of both steric and energetic effects reduces the available phase space, and hence the mobility, by the same amount for both regions. On the contrary, *β*-sheet mobility is not captured at all, probably because we do not consider in our analysis the non-local inter-strand interactions that are crucial for their stability.

### Structure refinement

The ability of exploring completely the conformation space of a limited fragment of a protein can be exploited for reconstructing or refining a small portion of a polypeptide structure. Let us suppose to have a putative fragment of a protein which has been roughly reconstructed by experimental methods: the hard degrees of freedom (bond angles and bond lengths) of this portion have correct values, whereas some of the torsional angles may not all be consistent with the allowed values of the Ramachandran’s plot. Given such initial configuration Γ_0_, our approach allows to modify exhaustively the soft degrees of freedom and to check if it is feasible to obtain a solution which is compatible with the standard Ramachandran’s plot.

To test this possibility, we first partition the Ramachandran’s plot in a grid of squares with size 2^°^ × 2^°^. By analyzing the databank Top500 formed by a non-redundant, specially refined set of 500 high resolution X-ray crystallographic structures of globular proteins [25], we consider as *good*, those bins which have a fractional occupancy higher than 0.04. We investigate if the condition of having *good* Ramachandran’s angles in a small fragment of a protein is a condition sufficient to reconstruct/refine the protein backbone. Therefore we randomly explored the conformational space of different portions of a protein. For each portion we store only the *acceptable* configurations, *i.e*. those configurations with good Ramachandran’s angles.

**Fig 11:**
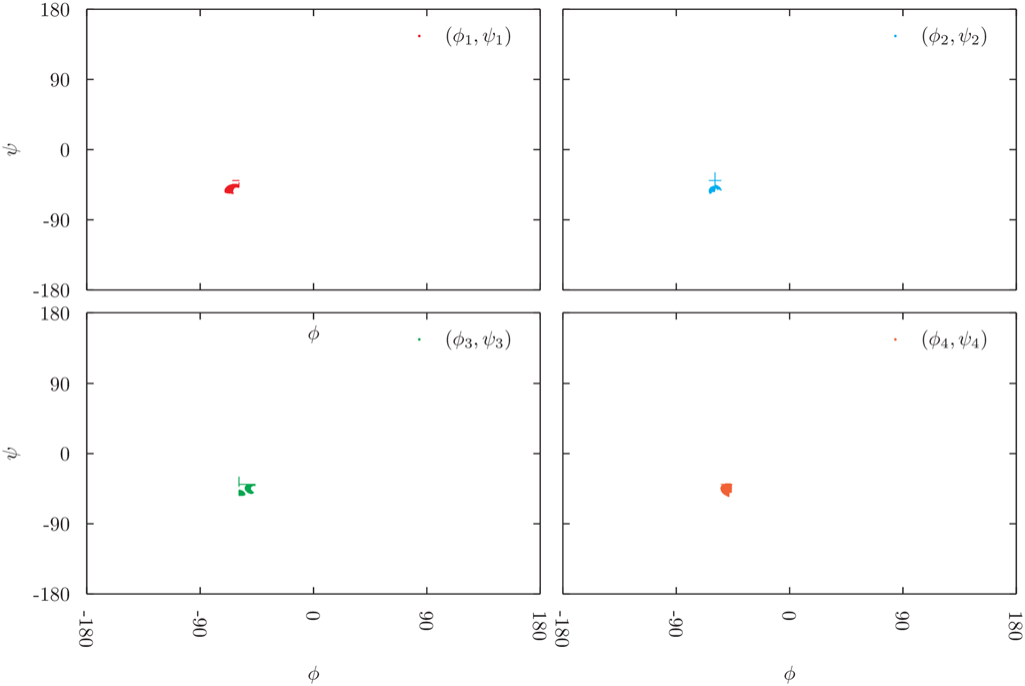
All acceptable Ramachandran angles obtained during the exploration of the solution manifold of a 5-residue-long *α* fragment. All points form a unique cluster, meaning that there is only one acceptable conformation of the helix that is compatible with the initial configuration. Thus, having fixed the first and the last bond, there is only one possible way to reconstruct the missing helix.

Different 5 residues long fragments were analyzed, from *α*, *β*, or coil structures (that are neither in *α* nor in *β*). Fig.s 11 and 12 show all the acceptable configurations that have been generated during the exploration of an *α*-helix and a *β*-strand portion, respectively. Each subplot shows the values sampled by a different pair of Ramachandran’s angles within the considered fragment. From Fig. 11, we can notice that all acceptable solutions generated from an *α*-helix structure lie in a very narrow region of the Ramachandran’s plot. The exploration of different *α*-helix regions confirms this finding and shows that usually about the 90% of the configurations that are compatible with the imposed constraints are acceptable, and hence shown in Fig. 11.

**Fig 12:**
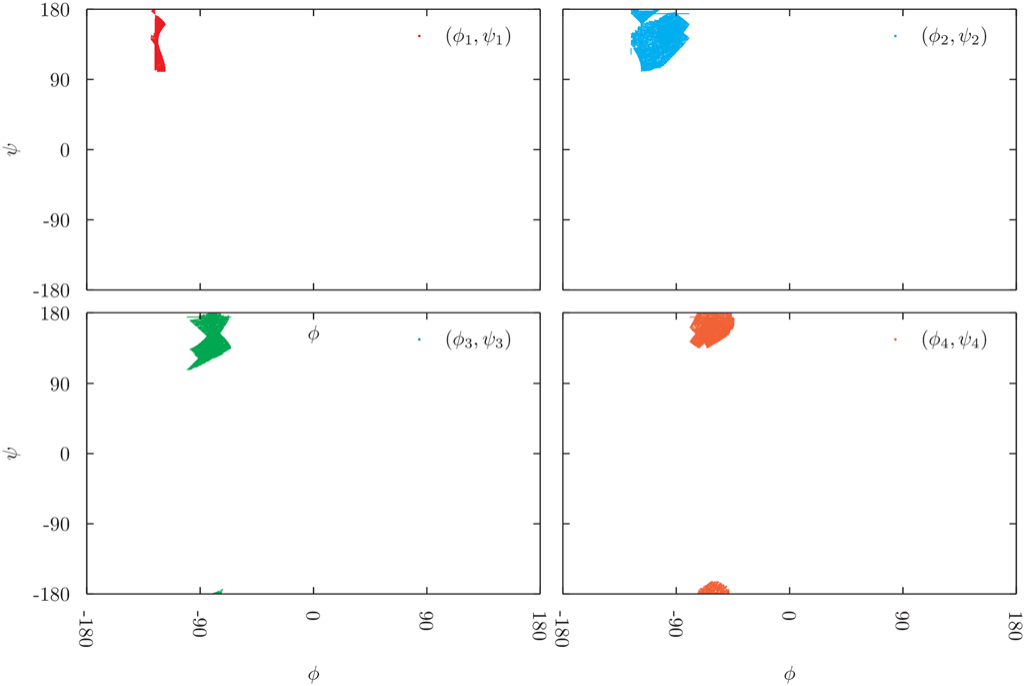
All acceptable Ramachandran angles obtained during the exploration of the solution manifold of a 5-residue-long *β* fragment. Points form different clusters, meaning that there are different acceptable conformations of the strand that are compatible with the initial configuration. It is not possible to safely reconstruct or refine a *β* fragment by only requiring the final configuration to have acceptable Ramachandran’s angles.

Going back to the original problem of refining a rough initial estimation of a protein portion, our results imply that, for an *α*-helix, it is easy to reconstruct a solution that is essentially unique, within very small changes of Ramachandran’s angles. Results are different in the case of *β*-structure; in such situation the algorithm works smoothly to find solutions but most of them are not in good regions of the Ramachandran’s plot (more than 90%) and those satisfying such constraints, the ones shown in Fig. 12, are more widely distributed in the good region. Therefore, a reconstruction starting by a *β*-like structure is feasible within our approach, but produces a broader range of possible solutions with respect to *α*-helical fragments. For both the *α* and the *β* fragments, in order to evaluate how exhaustive is our exploration strategy, the stored configurations were analyzed with a sophisticated cluster algorithm [26], checking that the conformational clusters found in each half of the search trajectories do not change.

Repeating the same analysis for coil fragments, it is found that only a very low percentage (usually below 1%) of configurations have good Ramachandran’s angles, whereas the acceptable configurations span all possible allowed values (data not shown). Under such circumstances, an exhaustive search of all possible acceptable solutions can be quite time consuming.

## Discussion

We introduced a novel technique that can be used to locally deform linear or cyclic polymer chain structures. Different kinds of degrees of freedom (torsional angles, bond lengths, bond angles or any arbitrary combination of these) can be used. There are no general requirements on the choice of the degrees of freedom: in the case of protein chains they can belong to the same residue as well as to residues that are far away from each other. In the general case, for the algorithm to work, it is necessary that at least seven different degrees of freedom are used.

The three-dimensional configuration of a linear chain is commonly described by using the cartesian coordinates of each monomer with respect to the same fixed frame of reference. Instead, in our algorithm we use the Denavit-Hartenberg (DH) convention [16, 17], that is very popular in robotics and has already been used by different authors [3, 11, 27–29] in order to describe a polypeptide chain. One advantage of the DH convention is its ability to readily describe a general disconnected subset of the bonds of the physical chain. This is useful if one is interested in varying in a concerted way degrees of freedom from disconnected chain segments, while keeping fixed the degrees of freedom in between. This occurs, for instance, when consecutive *ϕ*, *ψ* Ramachandran’s angles are chosen to be varied, whereas the torsional angle *ω* around the peptide bond is kept fixed [5]; in this case it is not necessary to include the peptide bond in the DH description (see Fig. 1).

In the simplest case, when all bonds included in the DH description are connected with each other, the DH variables have a well defined physical meaning. Since two consecutive bonds always share an atom, link offsets **d** are zero, and therefore we can interpret link lengths **r** as *bond lengths*, link twists *π* − *α* as *bond angles*, and joint angles *θ* as *torsional angles*. In the general case, some physical bonds may not be considered so that two consecutive bonds included in the DH description do not share an atom. When a disconnected bond is added, the link offset is different from zero, the link length and the link twist do not have a physical interpretation anymore, whereas the joint angle can still be interpreted as a torsional angle, albeit with an offset in its definition. As a consequence, torsional angles can always be included within the DH description as such, bond angles only if the previous bond in the DH description is not disconnected, and bond lengths if both the previous and the subsequent bond are not disconnected.

In general, the change of a single DH parameter is responsible for a global modification of the structure, *i.e*. a modification that, on average, affects a number of atoms proportional to the number of atoms of the chain. For instance the change *θ*_*i*_ → *θ*_*i*_ + Δ*θ*_*i*_ is responsible for a rotation around *ẑ*_*i*−1_ of all the atoms of the chain that belong to the bonds labeled with *j* > *i*; this configuration change is known as a pivot move. In order to locally update a configuration {**r**_0_, *α*_0_, **d**_0_, *θ*_0_} it is necessary to simultaneously change more than one DH parameter with the costraint that the remaining part of the chain is kept fixed. By using the DH description we were able to write a set of equations (see Eq. (9)) that describe the constraints and whose solutions correspond to the locally deformed configurations we want to compute. This set of equations is the central mathematical framework on which several other methods are based on [3, 11, 27–29]. The solutions derived by existing approaches are however limited to considering only torsional angles. Moreover, in order to compute a solution of the equations, existing techniques usually restrict their application to the study of particular geometries, such as the ideal Pauling-Corey geometry, or need to slightly modify a number of other degrees of freedom of the chain.

Our method generalizes existing algorithms by proposing a strategy that allows for a concerted modification of any arbitrary set of degrees of freedom of the chain while keeping *all* the other strictly fixed. The only requirement is that the number of selected degrees of freedom is greater than the number of linearly independent equations that define the constraints in Eq. (9), *i.e*. is greater than 6 in the non degenerate case.

All algorithms performing concerted local structural changes for a polymer chain roughly follow the same general strategy. First, a *pre-rotation* step is proposed, that is an update of a selected subset of *driver* pre-rotation angles among all the ones that will be eventually involved in the local move. If actually performed, the pre-rotation would generate an intermediate configuration *ξ* ^′^ corresponding to a global structural change. Then, the *post-rotation* step is performed, by explicitly finding the remaining post-rotation angles that satisfy the locality constraints.

The algorithm here proposed introduces a novel way to perform the pre-rotation step (see Fig. 3). Indeed, while other methods arbitrarily selects the driver angles and then generate the intermediate configuration *ξ* ^′^ by randomly perturbing them, our algorithm generates *ξ* ^′^ by moving from the initial configuration along a random direction in the tangent space to the manifold of the configurations compatible with the locality constraints. Thus, the pre-rotation step is already a change of all the degrees of freedom involved in the local move concerted in a way that is intrinsically driven by the geometrical properties of the manifold of explorable configurations.

The post-rotation step is then performed by using a root-finding algorithm to converge again to the manifold of correct configurations. Despite its simplicity, the root-finding approach is effective since it takes advantage of the fact that the intermediate configuration *ξ* ^′^ is already a good approximation to the correct solution as well as of the fact we can restrict the root-finding algorithm to search for a solution by moving within the space orthogonal to the manifold at the initial configuration. The orthogonal space can be efficiently computed based on the knowledge of the tangent space already needed in the pre-rotation step. Restricting the search of the solution within the orthogonal space also ensures that the solution searched for in the post-rotation step is unique, for small enough pre-rotation moves, providing at the same time a simple way to compute the probability of the backward transition and thus to enforce detailed balance in a Monte Carlo simulation.

The possibility to numerically compute the derivatives as in Eq. (11) is not only useful to determine the basis vectors for the tangent space to the manifold of chain configurations compatible with the locality constraints, but also to obtain any directional derivative on it of scalar functions that depend on chain configuration such as, for instance, potential energy functions. Notably, 300–400 concerted moves per second can be performed on a single core 2.0 Ghz processor in the present implementation. This makes the efficiency of our general numerical methodology not so distant from the one reported in [13], 2000 loop closure solutions per second, with an analytic-based strategy that relies on a specific choice of the torsional angles to be modified.

Importantly, once the difference between forward and backward probabilities is taken into account, the usage of orthonormal basis vectors in both the tangent and orthogonal spaces ensures that the space of DH parameters involved in the local move is sampled uniformly in a Monte Carlo simulation, if no other reweighting is employed in the acceptance/rejection test. This is shown in Fig. 4 for the simple case of DH parameters that correspond to torsional angles, that are indeed expected to display a uniform distribution at equilibrium in the absence of any interaction. Thus, at variance with existing algorithms, we do not need to perform, for further reweighting, the time-consuming calculation of the Jacobian factor due to the solution of the post-rotation step [7].

For DH parameters that correspond to bond lengths and bond angles, that are not expected to have uniform distributions at equilibrium, no reweighting is again needed, provided that the locality constraints and the corresponding manifold of possible chain configurations are defined in terms of simply modified variables that are instead expected to be uniformly sampled.

Moreover, a simple rescaling of uniformly sampled variables used in the local move maintains their sampling properties while allowing to tune the relative fluctuations of the non rescaled variables. This could be useful in dealing with polypeptide chains, when variables originally related to bond lengths and bond angles are expected to fluctuate much less than torsional angles. The need of further reweigthing is again avoided due to the proper exploitation of the intrinsic geometrical properties of the manifold of correct configurations, as defined in terms of the rescaled variables. However, as shown in Fig. 5, the tuning of relative fluctuations by means of variable rescaling is possible only for manifolds with dimension at least two. Moreover, Fig. 5 shows that the effect the rescaling of one variable induces on the other variables, through the coupling with local manifold geometry, is in practice negligible. As a consequence, we may expect that the number of variables whose fluctuations can be independently tuned by rescaling is given by the manifold dimension minus one. Consistently, as shown in Fig. 6, for unidimensional manifolds the relative fluctuations of the different variables involved in the local move are in itself dictated by manifold geometry and are not affected by rescaling.

Instead, if DH parameters that do not have a physical interpretation are involved in the local move, it is not possible to go simply back to the case of uniform sampling. It would be necessary to take into account the expected non uniform sampling of all bond lengths and bond angles related to the unphysical DH parameters by proper reweighting factors.

We showed different possible applications of the technique that we introduced.

A first application is an efficient scheme to explore the whole configurational space of small fragments of a polypeptide backbone or of other chain structures that are compatible with locality constraints. We demonstrated the concept, first, in the simplest case of a protein fragment where the degrees of freedom involved in the local move are 7 consecutive *ϕ*, *ψ* Ramachandran’s angles along the polypeptide backbone. In that case, all possible configurations to be explored lie on a one-dimensional manifold embedded in a periodic 7-dimensional space, whose projection in a 3-dimensional space is shown in Fig. 7. Since our technique relies on the computation of the tangent space to the manifold, in order to define the pre-rotation step, it is both straightforward and efficient to stride along the manifold following the same direction until the starting configuration is revisited.

For higher-dimensional manifolds the systematic exploration of all possible configurations is a difficult task. We employed the cyclo-octotetraene cyclic molecule as a toy model, by considering all its 8 torsional angles as degrees of freedom. The manifold of possible configurations (in the special case of a cyclic closed chain these are *all* possible configurations) is a bidimensional one in an 8-dimensional space. We used the strategy of dividing the exploration in separate one-dimensional trajectories that are tracked along the same direction until the initial configuration is recovered, as in the previous case. The whole manifold can be recovered in this way, by changing the initial configuration and the subset of 7 torsional angles to be varied along a given one-dimensional trajectory. The resulting manifold is shown as a projection in a 3-dimensional space in Fig. 9 and some representative conformations are shown in Fig. 8. For higher-dimensional manifolds, the usage of more sophisticated sampling techniques, such as generalized ensemble Monte Carlo methods [30] or metadynamics [31], may well be more efficient.

It is important to observe that our technique relies on the previous knowledge of a configuration already compatible with the locality constraints, and the loop closure problem is solved only at a local level, in the post-rotation stage (see Fig. 3). In the classic loop closure problem, instead, one is given the task of reconstructing ‘ab initio’ a missing portion of a linear chain. It is then easy to do it by using ‘wrong’ values for, say, one bond length and one bond angle. The hard problem of finding a configuration with the ‘right’ values, then, could be in principle recast, within the framework of our technique, as the problem of the searching for a subset of configurations with the ‘right’ values within a manifold suitably chosen where the bond length and the bond angle to be fixed are among the degrees of freedom that are allowed to change. While the actual implementation of the above sketched strategy is beyond the scope of the present paper, it provides a context where the exploration efficiency demonstrated by our technique could prove extremely useful.

The other applications of our technique that we investigated are more directly related to the local distortion properties of protein chain structures, when only backbone heavy atoms are considered.

First, we introduced the notion of *local backbone volume*, as the volume spanned in the 3*N*-dimensional Cartesian space by all configurations that can be adopted by a protein segment, compatibly with the locality constraints, as the degrees of freedom involved are changed on the corresponding manifold. As a consequence, the local backbone volume may strongly depend on the choices made for both the constraints and the degrees of freedom. Locality constraints can differ a lot depending, for example, on the secondary structure content of the considered protein chain segment.

It is natural to relate the volume at disposal for local concerted movements to the mobility of residues, so that the higher the local volume the more mobile the residues. Indeed, the local backbone volume computed for different protein chains, in the simplest case of 7 consecutive *ϕ*, *ψ* Ramachandran’s angles, allows to easily recognize *α*-helices as the most locally rigid portions of proteins. In the case of an all-*α* helical protein, a quantitatively good matching can be performed between the local volume profile and the residue mobility profile resulting by different NMR structural models of the same protein (see Fig. 10). This is a non trivial result, since the local backbone volume is a geometrical feature that does not take into account any interaction or excluded volume effect. Consistently, *β*-strands are not identified as locally rigid segments in our approach (see Fig. 10), since, at variance with *α*-helices, they need to be stabilized by hydrogen bonding to a nearby strand.

Second, we tried to assess the following point: How many realistic protein structures do exist that are compatible with a given locality constraint? This is a central issue in structure refinement, when the task is often to improve over an existing non realistic configuration of a protein segment. In practice, we start from a real protein segment configuration and we simply use our technique to perform a thorough exploration of the manifold of possible solutions compatible with the locality constraints. We look for standard values of the Ramachandran’s angles *ϕ* and *ψ* to filter realistic structures. Again, the locality constraint, and thus the answer to the raised question, crucially depend on the secondary structure of the chosen protein segment.

We use a state-of-the-art cluster analysis to make sure that the exploration of the manifold was completed, when no new clusters are observed. Consistently with the local backbone volume analysis, we observe that if the initial segment has an *α*-helical structure, most of generated configurations are realistic, the latter are all *α*-helical ones and span a very narrow region in the Ramachandran’s plot (see Fig. 11). If the initial segment has a *β*-strand structure, the fraction of generated configurations decreases, whereas all realistic configurations found are in the *β*-strand region of the Ramachandran’s plot, spanning a wider region (see Fig. 12). If the initial segment has no secondary structure, the generated configurations essentially span the whole Ramachandran’s plot and the fraction of realistic conformations is very small. Based on pure geometrical properties, a helical structure is quite easily refined, a strand segment is less easily refined, a loop coil region is not quite easily refined.

Our aim here is to show how the efficiency of our local exploration technique can be easily employed in the context of protein structure refinement; systematic results could be obtained by testing, within the same approach, initial segments as helix or strand ends, or hairpin turns. More importantly, a bias can be easily incorporated in the sampling of the manifold of possible solutions, according to a potential energy function, or to a general scoring function, or to a measure of consistency with known experimental data, such as electron density maps for X-rays diffraction experiment on protein crystals.
